# The Learning Curve of Pure Retroperitoneoscopic Donor Nephrectomy

**Published:** 2017-11-01

**Authors:** B. C. Pal, P. R. Modi, S. J. Rizvi, R. Chauhan, S. Kumar, R. Nagarajan, D. Kaushal, V. B. Kute, H. L. Trivedi

**Affiliations:** 1 *Department of Urology and Transplantation Surgery, Institute of Kidney Diseases and Research Centre and Institute of Transplantation Sciences, Civil Hospital Campus, Ahmedabad – 380016, Gujarat, India*; 2 *Department of Nephrology and Transplantation Medicine, Institute of Kidney Diseases and Research Centre and Institute of Transplantation Sciences, Civil Hospital Campus, Ahmedabad - 380016. Gujarat, India*

**Keywords:** Laparoscopy, Nephrectomy, Renal transplantation, Learning curve, Tissue donors, Safety

## Abstract

**Background:**

Retroperitoneoscopic donor nephrectomy (RDN) is a well-established modality for the procurement of kidneys for renal transplantation. However the learning curve of pure RDN is not yet defined. Defining the learning curve will help in proper mentorship of the new donor surgeons besides providing safety to the donors.

**Objective:**

To define the learning curve of pure RDN.

**Methods:**

We analyzed the prospectively collected data of 102 voluntary kidney donors who underwent RDN by a single surgeon between August 2012 and April 2015 at our center. The donors were classified into group A (1–34), group B (35–68), and group C (69–102) according to the chronological order of their surgery. Left RDN was performed in 28 (82%), 25 (74%), and 28 (82%) donors of group A, B, and C, respectively. Right RDN was performed in 6 (18%), 9 (26%), and 6 (18%) donors of group A, B, and C, respectively. The clinical data were analyzed for each group.

**Results:**

Statistically significant difference was observed for the mean operative time (p<0.01) and warm ischemia time (p<0.04). The operative time remained around 200 minutes after the initial 35 cases.

**Conclusion:**

The learning curve of pure RDN was 35 cases, although the mastery requires more number of cases to be performed.

## INTRODUCTION

Donor nephrectomy is a demanding surgery in a way that it puts the donor health at risk without any benefit to them. Since its introduction in 1995, laparoscopic donor nephrectomy has been established as the standard of surgery for the procurement of kidneys for transplantation [1]_._ It improves the morbidity and shortens the convalescence period. Retroperitoneoscopic living donor nephrectomy has the additional intraoperative advantage of direct and early access to the renal pedicles besides avoiding any potential injury to the intraperitoneal organs [2, 3]. As more centres will take up this procedure because of increase in burden of the live-related transplantation program across the globe, it is of paramount importance to define the learning curve of this procedure so that new donor surgeons get a proper mentorship and at the same time the interest of the donors are safeguarded. However, literature on the learning curve of the pure retroperitoneoscopic donor nephrectomy (RDN) is lacking. In this study we tried to define the learning curve of pure RDN.

## PATIENTS AND METHODS

We analyzed prospectively collected data of 102 voluntary kidney donors who underwent RDN by a single surgeon between August 2012 and April 2015 at our institute. The surgeon was well trained in retroperitoneoscopic surgeries and started doing RDN independently after observing 20 cases.

The suitability of the donor was evaluated by an interdisciplinary team comprising of a urologist, nephrologists, an anesthetist, a transplant coordinator, and a psychologist.

After a complete history and physical examination, the required laboratory investigations and a basic radiological workup in the form of sonography of the abdomen and KUB, donors were subjected to diethylene triamine pentaacetic acid (DTPA) renal scan for the functional assessment and computed tomography (CT) renal angiography for the anatomical assessment of the vasculature and the draining system.

The laterality of the surgery was decided based on the presumption that the better kidney remains with the donor. If both kidneys had equal function, the kidney with simpler vascular anatomy was procured.

Surgical Technique

For left RDN (LRDN), the donor was secured in right lateral decubitus position and the table was flexed to open the space between the rib cage and the iliac crest. A 1.5-cm incision was made in the mid-axillary line below the rib. A double gloved finger balloon was used to create the retroperitoneal space. A 10-mm laparoscopic port was placed, brought near just inside the edge of lumbodorsal facia and fixed. Pneumoretroperitoneum was created with a pressure of 15-mm Hg. Under vision, additional 10-mm and 5-mm ports were placed at renal angle and anterior axillary line, respectively. Gerota’s fascia was incised. The ureter gonadal complex was dissected together. As the flimsy layer of loose areolar tissue between the psoas and perirenal fat was dissected medially, the hilum was reached where the pulsation of renal artery was observed. Lumbar vein complex was found in the vicinity of renal artery. It was dissected and secured with LigaSure™ (Valleylab, Tyco Healthcare, Boulder, CO, USA). The renal artery and the posterior aspect of the renal vein were dissected. The posterior pannus of the perirenal fat was excised from the upper to lower pole. Anteriorly, the flimsy loose areolar tissue between the renal capsule and the perirenal fat was freed and the kidney was mobilized. The adrenal gland was identified anterior to the artery with its distinct lemon-yellow color. The adrenal vein was identified. Having it as a guide, the anterior surface of the renal vein was dissected. Adrenal vein was controlled with ligasure. The lower pole of the kidney was mobilized and the insertion of the gonadal vein was seen with the renal vein. The gonadal vein was controlled with ligasure. The ureter was clipped distally over the bifurcation of common iliac artery and cut. A modified Gibson incision of 7–8 cm was made for retrieval. The renal artery and vein were divided with endoshears distally after applying two Hem-o-lok^®^ clips (Weck Closure Systems, Research Triangle Park, NC, USA) on them, respectively. Lubricated palm of the hand was introduced through the retrieval incision and the kidney was glided over psoas and brought out. The excised fat was removed. The kidney was perfused with cold HTK solution in ice slush. The retrieval wound was closed in layers using vicryl 2-0 (polyglactin 910). The fossa was inspected for any minor bleeding or lymphatic ooze which was taken care of. The skin was closed by absorbable suture.

For the right RDN (RRDN) the dissection was done in the similar fashion. An additional 12-mm port was placed 1 cm above the iliac crest on the lateral aspect of paraspinal muscles for introducing the stapler. After the artery was doubly clipped and cut, the kidney was retracted up to slightly tent the inferior vena cava (IVC). Endo-TA (Auto Suture, US Surgical, Norwalk, CT, USA) stapler was applied over the IVC below the renal vein. The renal vein was divided distal to the staple line giving a cuff of IVC [4]. 

The studied 102 donors were classified into group A (1–34), group B (35–68), and group C (69–102) in the chronological order of their surgery. The clinical data were obtained for each group. Preoperative characteristic of age, sex, BMI, multiple arteries, venous anomalies and intraoperative characteristic of total operative time (OT) (defined as the time from the skin incision to the skin closure), warm ischemia time (WIT) (defined as the time from clamping the renal artery to the starting of the cold HTK perfusion), blood loss, and intraoperative and postoperative complications were analyzed.

Statistical Analysis

The data were analyzed with SPSS^®^ for Windows^®^ ver 20. Continuous variables were expressed as mean±SD. One-way ANOVA and Kruskal-Wallis test were used to compare studied groups. χ^2^ and Fisher Exact tests were used to compare categorical variables. A p value <0.05 was considered statistically significant.

## RESULTS

Table 1 summarizes the characteristics of the donors in groups A ,B, and C. The distribution of age, sex, and BMI did not differ significantly among the groups. There mean blood loss was not significantly different among the three groups. However, one of donors in group A had significant blood loss because of arterial injury.

**Table 1:** Demographic and clinical data of the 102 donors

Variables	G-A (1–34)	G-B (35–68)	G-C (69–102)	p value
Age (yrs)	43.3±10.1	47.3±9.3	45.7±11.3	0.28
Sex				
Male	12 (35%)	9 (26%)	11 (32%)	0.73
Female	22 (65%)	25 (74%)	23 (68%)	
BMI (kg/m^2^)	24.0±4.2	24.7±4.5	25.2±5.5	0.60
Kidney laterality				
Left	28 (82%)	25 (74%)	28 (82%)	0.58
Right	6 (18%)	9 (26%)	6 (18%)	
Number of arteries				
1	31 (91%)	28 (82%)	23 (68%)	0.05
2	3 (9%)	6 (18%)	11 (32%)	0.05
Venous Anomaly	2 (6%)	4 (12%)	3 (8.82%)	0.56
Circumaortic	2 (6%)	1 (3%)	1 (3%)	0.69
Retroaortic	0 (0%)	2 (6%)	2 (6%)	
Double IVC	0 (0%)	1 (3%)	0 (0%)	
OT (min)	295.6±42.8	235.6±20.2	216.2±42.7	<0.01
WIT(sec)	231.8±96.2	201.7±45.4	187.7±68.0	<0.04
Blood loss (mL)	84.7±136.7	52.2±25.5	44.4±26.0	0.11
Intraoperative complication	2	0	0	
Arterial injury	1	0	0	
Venous injury	1	0	0	
Postoperative complication	0	1	0	

**Figure 1:**
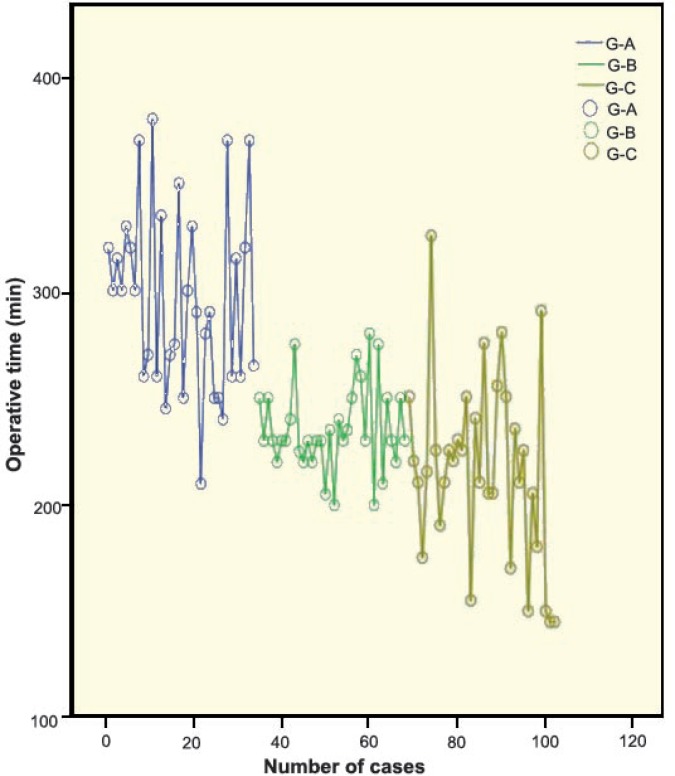
Cluster line graph showing the operative time in studied groups

The mean OT decreased consistently with passage of time (Table 1, Fig 1). When group A was compared with group B, the difference in OT was 60 min (p<0.01). When group B was compared with group C, the difference was only 19 min (p<0.02). Similar significant differences were observed for WIT.

Two cases in group A required conversion to open surgery because of vascular mishaps. Kidney transplantation was successfully carried out in recipients of the two donors. One donor in group B had a large retroperitoneal lymph collection post-operatively. It was treated conservatively with octreotide (0.1 mg) thrice daily for two weeks.

Details of the recipient outcome are presented in Table 2. Significant difference was observed in day one serum creatinine (Table 2).

**Table 2:** Recipient data for the corresponding donors. Values are mean±SD.

Serum Creatinine at	G-A (1–34)	G-B (35–68)	G-C (69–102)	p value
1 day	2.90±1.02	2.72±0.83	2.05±1.47	<0.01
1 month	1.17±0.28	1.17±0.29	1.28±0.57	0.43
1 year	1.24±0.35	1.24±0.44	1.34±0.71	0.72

## DISCUSSION

The statement “do not further harm” is apt for the donor surgery. Ample literature is available on RDN. However, literature on its learning curve is lacking. Chin, *et al*, described a learning curve of 150 cases for laparoscopic donor nephrectomy [5]. In their study the OT decreased by 87 min after the initial 150 cases. Besides, the complications reduced from 86.2% in the initial 150 cases to 10.3% in subsequent 150 cases. In the study conducted by Tokodai, *et al*, the learning curve decreased over the first 30 cases and began to plateau thereafter [6]. Dols, *et al*, showed that hand-assisted RDN had a shorter OT compared with laparoscopic donor nephrectomy [7].

In our study, the mean OT reduced significantly in groups B and C in comparison with group A (p<0.01). Most of the cases in group A required more than 300 min for the surgery. The difference in mean OT observed between groups A and B was 60 min (p<0.01); the difference between groups B and C was only 19 min (p<0.02). Although the difference was still significant the difference got smaller by passage of time between groups B and C. The variance in OT was high, particularly in groups A and C because of the complexity of the cases. Similar changes were observed in WIT.

Several studies have shown a conversion rate of 1.6%–2.8%, which is observed in the early part of the learning curve [8-11]. We had similar observation and witnessed two open conversions because of vascular mishaps in the first 34 cases (group A).

We observed that the critical steps in the learning were in dealing with (a) lumbar vein complex, (b) the perirenal and pararenal fat, (c) the lymphatic tissue dissection between the renal artery and vein, (d) controlling the adrenal vein from the anterior aspect, (e) managing retroperitoneal space restriction, and (f) managing peritoneal rents, if any, occur during the course of the surgery.

The lumbar veins usually pass near the origin of the renal artery and drain into the renal vein posteriorly. Their anatomy has variations, as described by Li, *et al* [12]. Dissection of lumbar veins requires utmost care and precision to avoid injury to them, which can lead to significant bleeding. We had no injury to lumbar veins in our study. In one case, the renal vein was mistaken with lumbar vein as it was seen posterior to the artery and was clipped and cut requiring immediate open conversion. Review of the CT angiography showed that the course of the renal artery after its origin was anterior to the renal vein. 

In retroperitoneoscopic surgery the space is limited. In obese donors, the limitation is higher because of the thick pannus of pararenal and perirenal fat. The “gas dissection effect” of the CO_2_ allows for dissection in the avascular plane between the perirenal fat and the renal capsule [13]. The posterior perirenal and the pararenal fat should be excised. This helps in creating more working space near the kidney. Additionally, the hanging fat can be a nuisance as it can touch the camera tip, which can hamper the vision. Frequent removal of the scope and its cleaning increases the OT. Furthermore, the smoke produced by the activation of the cautery in the limited space makes the vision foggy. Using the laparoscopic suction cannula is helpful, as it helps in sucking the smoke besides helping in retraction. 

Sometimes, a small peritoneal rent leads to significant loss of retroperitoneal space. This is dealt by putting a veresse needle in the peritoneal cavity. If this manoeuvre does not work, the opened peritoneal edges are pinched together by applying few Hem-o-lok^®^ clips.

Dissection of the lymphatic tissue between the renal artery and renal vein from the posterior aspect occasionally becomes difficult, especially in donors who smoke where the lymphatic tissue is “sticky.” Baring the aortorenal junction from the lymphatic tissue helps in freeing the intervening lymphatic tissue between the artery and vein completely from the posterior aspect. Identification of the adrenal vein is facilitated by making the upper pole completely free so that the renal artery can be seen from anterior aspect, when the upper pole is retracted inferiorly. The adrenal gland is identified with its lemon-yellow color; it is dissected off the dull-yellow color fat over the kidney. This dissection leads to the adrenal vein.

The ureter is clipped and cut when the dissection is complete, as dissection and use of energy after cutting the ureter has a risk of injuring the “free” ureter. In our study the surgeon had a dominant left hand, which was an advantage when performing RRDN. Dissection of lymphatics to bare the IVC during the procedure was more precisely performed with the left hand.

In our study we used Hem-o-lok^®^ clips to control the vascular pedicle. It is an institutional protocol at our place to use the clips and in over 2000 RDNs performed at our institute, we have not encountered any vascular mishap due to clip failure. We and others have described the safe use of Hem-o-lok^®^ clips in RDN before [14, 15]. 

In the recipient, day one serum creatinine clearance was better in group C. However one-month and one-year serum creatinine levels did not differ statistically among the groups. The limitation of our study was that it was based on the analysis of performance of a single surgeon. Moreover, complex cases were also operated in the early part of the learning curve where the OT tended to be more.

In conclusion, pure RDN had a learning curve of 35 cases although to achieve mastery of the technique operation on more number of cases would be necessary. The critical steps in learning were in dealing with the retroperitoneal space, the lumbar vein complex, the perirenal and pararenal fat, the intervening lymphatic tissue between the renal artery and vein, and controlling the adrenal vein from the anterior aspect.
